# How does eye care seeking behaviour change with increasing age and visual impairment? Intersectional analysis of older adults in the Indian Sundarbans

**DOI:** 10.1186/s12877-020-1438-y

**Published:** 2020-02-18

**Authors:** Debjani Barman, Manasee Mishra

**Affiliations:** IIHMR University, Jaipur, India

**Keywords:** Visual impairment, Dependency, Ageing, Older adults, Intersectionality, Qualitative sampling, India, Gender

## Abstract

**Background:**

Visual impairment disproportionately affects people in the low-income countries. A high proportion of visual impairment can be prevented or cured. Yet, care seeking for eye health is restricted for women and older adults. This article uses the intersectionality approach to understand how eye care seeking behaviour changes in men and women with increase in age and visual impairment in a poor and underserved region of India. It brings forth the commonalities and differences between the various groups.

**Methods:**

The article is based on qualitative data. Persons aged 50 years and more are categorized into young-old, middle-old and old-old. Men and women with low vision/ high visual impairment have been selected from each of the three age groups. In-depth interviews have been carried out with 24 study participants. Data saturation has been attained. The JHPIEGO Gender Analysis Framework underpins the study. The narrative data has been coded in NVivo 10 software.

**Results:**

Various symptoms are associated with visual impairment. The young-old with low vision do not report much difficulty due to visual impairment. Study participants with high visual impairment, and in the older age groups do. Difficulty in the discharge of regular chores due to visual impairment is rarely reported. Impaired vision is considered to be inevitable with advancing age. Care seeking is delayed for eye health. Typically, outpatient care from nearby health care facilities has been sought by men and women in every group. Inpatient care is limitedly sought, and mostly restricted to men. Eye care seeking behaviour changes among men with increase in age and visual impairment. Women consistently seek less care than men for both outpatient and inpatient eye care. Study participants of both genders become dependent with increasing age and visual impairment. Traditional patriarchal privileges enjoyed by men (such as mobility and economic independence) decrease with age. The vulnerability of women gets compounded with time.

**Conclusions:**

The article presents a granulated understanding of eye care seeking behaviour among older adults in India. Such differentials need to be taken cognizance of in programmes promoting universal access to health care. Existing conceptualizations on access to health care need to be revisited.

## Background

Eye health can be largely secured by timely preventive and care seeking measures. It is estimated that, globally, of the 7.3 billion people alive in 2015, more than 440 million people had compromised eye health status. Of these, 36.0 million were blind, 216.6 million had moderate to severe visual impairment and 188.5 million had mild visual impairment. The proportion of women was higher in every category [1]. Approximately 90.0% of the visually impaired in the world live in low-income settings and 82.0% of the people living in blindness are aged 50 years and more [2]. Most cases (80.0%) of visual impairment can be prevented or cured. Uncorrected refractive errors are the main cause of moderate and severe visual impairment in the world and cataract is the leading cause of blindness in low- and middle- income countries (ibid). In a review of 14 studies in Asia, it was found that cataract and uncorrected refractive errors were the most common among age related eye diseases [3]. India is expected to ‘turn into a rapidly ageing society in the coming decades’ [4]. A recent survey in the country showed that 70.0% of the study population aged 50 years and more have impairments in either near or distance vision [5].

Access to health care in low- and middle- income countries incorporates the dimensions of quality, geographic accessibility, availability, financial accessibility and acceptability of services [6]. Women’s access to health care can be particularly compromised because of their gendered existence. Lewallen and Courtright note that the cataract surgical rate is higher for men in developing countries. Some reasons for such gender differentials include the high costs involving transportation to the hospital, loss of wages and expenses incurred during the stay in the hospital. The authors add that poor rural women usually have less disposable income or control of finances compared to men. Since cataract surgery requires transport to a hospital, women are less likely to seek it as it would entail travelling outside their village for the service [7]. Lower status in the family, restricted public mobility and lack of control over economic resources are reasons due to which women have lesser access to eye care services across the world [8].

India has pluralistic health systems. Health care is provided by the government and private players. The country has an inequitous and fragmented health care market with gross rural-urban disparities. Unqualified health care providers also provide care in the country, especially in areas inadequately served by the formal health care providers. The coverage of insurance is limited. Typically, high out-of-pocket expenses are borne for seeking health care. For a country of sub-continental proportions, there are pan-Indian gender differentials in access to health care [9].

Health care seeking is contingent upon timely deployment of material and non-material resources. Gender and age are widely regarded as disadvantaging identities in social science literature on health. However, personal identities, material lives and health are governed by a range of intersecting characteristics. The location of the individuals and groups at the intersection of societal structures and processes shapes one’s experiences. The intersectionality approach is being increasingly used in health systems research to better understand inequality where social categories such as gender, age, class and (dis) ability are considered as ‘mutually constituted and intersecting in dynamic and interactive ways’ [10]. This article adopts the lens of intersectionality to examine how the characteristics of gender, age and visual impairment intersect in the seeking of eye care in a disadvantaged region of India. The methodological approach and the findings in this article are novel, capturing as they do the dynamic and combined effects of multiple characteristics on eye care seeking behaviour. The article presents a case for looking at the needs of older adults in a nuanced manner.

## Methods

### Aim, design and study setting

This article is drawn from a larger study carried out to understand the effects of the intersection of gender with other identities/socio–demographic characteristics on eye care seeking behaviour among persons aged 50 years and more. The study is located in the Sundarbans region of India. The Sundarbans is mostly a deltaic region marking the confluence of the river Ganges with the Bay of Bengal. Its unique topography is spread across two countries (India and Bangladesh). It is a region of climatic uncertainties. As per the 2011 Census of India, the Indian Sundarbans is home to 4,426,259 people [11]. They reside in 54 out of the 106 islands in the Indian Sundarbans [12]. The region is predominantly rural and historically an impoverished and underserved area. Qualitative fieldwork was carried out in two administrative blocks of the Indian Sundarbans. The blocks are located 110 km apart from one another. The names of the blocks have been anonymized for ethical reasons.

Cases of ageing men and women with visual impairment were drawn from a quantitative dataset of the larger study. As per the National Programme for Control of Blindness, Government of India, visual impairment is classified into five categories graded in terms of severity [13]. The five categories are: normal vision, low vision, economic blindness, social blindness and one eye blindness. 1 During the quantitative data collection phase, a team of optometrists had carried out eye testing to identify the level of visual impairment of the ageing study participants and had classified each person into one of the five categories. Such a list of visually impaired persons (*n* = 442) provided the sampling frame for the qualitative ethnographic fieldwork. The mean age of the visually impaired persons in the quantitative dataset was 67 years. A majority of them were illiterate and economically dependent. In order to understand intersectionality, the qualitative sample was drawn from this quantitative dataset. The qualitative research principles of purposive selection and extreme case sampling were adopted for the purpose. Qualitative ethnographic fieldwork was carried out after the quantitative data collection was over.

### Characteristics of study participants

Three characteristics of the study participants have been taken into consideration for inclusion in the sample. These are gender, age and visual impairment. Cases of low vision and economic/ social/ one eye blindness were selected from within each such intersecting group of gender and age. 2A study participant was either a man or a woman, belonging to one of the three age groups of young-old (50–64 years), middle-old (65–74 years) or old-old (75 years or more), and with either low vision or high (economic/ social/ one eye blindness) visual impairment. Thus, there were 12 groups of study participants, members of each group belonging to a particular gender, age group and level of visual impairment (Table 1). Twenty four men and women were interviewed. Hindus and Muslims were represented in almost equal proportions in the sample.

**Table 1:** Qualitative Sampling Scheme

Visual Impairment	Young-Old (50–64 years)		Middle-Old (65–74 years)		Old-Old (75 years or more)	
	Men	Women	Men	Women	Men	Women
Low Vision	2	2	2	2	2	2
High Visual Impairment (economic blindness, social blindness or one eye blindness)	2	2	2	2	2	2
Total (24)	4	4	4	4	4	4

### Process of data collection

Both authors are females and hold PhD degrees. Both held faculty positions in a university at the time of the study. They have received professional training on qualitative research and have carried out several qualitative studies. Qualitative ethnographic fieldwork was iteratively conducted in two phases in the two study sites during the period December 2015–August 2016. First, face to face in-depth interviews were conducted by the lead author with the study participants. An in-depth interview guide had been developed by the authors for the purpose. It contained an indicative list of questions and probes. The guide had been pilot tested. Interviews were carried out in the homes of the study participants and in their native language. Prior to the interviews, relationship with the study participants had already been established during the optometric tests and the quantitative data collection. Study participants knew about our institutional affiliation and contact details. They knew that we were carrying out research and our reasons for doing it. They knew that the lead author was in charge of the study. They had been informed about these matters. Privacy was largely maintained, with other persons being rarely present during the interviews. The average duration of the interviews in this phase was 1 h and 20 min.

There were no refusals to participate in the study during the face to face in-depth interview phase. After a gap of some months, follow up interviews were conducted telephonically with 12 of the study participants. The telephonic interviews were primarily carried out to ascertain that data saturation had been attained for the various domains of information, and that no new aspects emerged. One study participant refused to participate during the telephonic interview phase. The reason for refusal is not known to us. No inconsistencies emerged in the narratives of the study participants during the telephonic phase of the fieldwork. Fieldnotes were maintained by the researchers throughout the fieldwork and a record of the memos was kept.

### Analysis

All in-depth interviews were audio recorded. The recordings were transcribed verbatim. The transcripts could not be shared with the study participants. Coding was undertaken in NVivo10 software. We adopted hybrid coding using a mix of deductive and inductive codes [14]. Gender analysis has been defined as ‘a systematic methodology for examining the differences in roles and norms for women, men, girls and boys; the different levels of power they hold; their differing needs, constraints, and opportunities; and the impact of these differences in their lives’ [15]. The JHPIEGO Gender Analysis Framework (GAF) [15] provides an analytical underpinning for this study. The framework has four domains, viz., access to assets; beliefs and perceptions; practices and participation; institutions, laws and policies. Power pervades the four domains. It determines access to and control of assets and decisions ‘over one’s body’. Ours being a community-based study, the deductive codes in the coding schema were primarily informed by pertinent concepts in GAF in the three domains of access to assets; beliefs and perceptions; and practices and participation. The inductive codes were data driven, primarily descriptive in nature, and as suggested by the narrative data. Related inductive codes were grouped together under the corresponding deductive codes to form coding categories. The major coding categories were perceptions about visual impairment; daily life and dependency; health status and health care seeking.

Two researchers carried out the coding in a complementary manner. A third person regularly checked the emerging coding structure to ascertain its appropriateness, and the assignment of appropriate code(s) to any given piece of narrative data. The coding tree was thus a continually evolving one and agreed upon by the researchers. The NVivo10 qualitative dataset for the study is rich in variety and depth, with concepts in GAF analytically underpinning the study. The dataset has a total of 1958 nodes and the coding structure reaches up to level 12 for the particularly deep mother nodes. The constant comparative method [16] has been used to identify patterns within and across the 12 groups of study participants. Quotations reported in this article are usually English translations of the statements made in the native language. All quotations have been italicized. For reasons of confidentiality, only the gender, age group and level of visual impairment of the concerned study participant are mentioned alongside the quotation. The themes have been derived from the data and there is consistency between the data presented and the findings. The major themes have been presented as sub-sections in the section on ‘Results’. Finer details and patterns have been teased out within each theme. The study participants have not provided feedback on the findings. The various aspects of the qualitative methodology adopted in the study are reported as per COREQ guidelines [17]. The filled up COREQ checklist is annexed (Additional file 1).

## Results

### Perceptions of visual impairment

References to eye health feature in the narratives of every study participant. Study participants in all groups associate various symptoms with their visual impairment. However, the narratives are least dense for the group of young-old with low vision. Visual impairment is believed to occur with advancement in age and does not particularly concern the young-old. Persons beyond the age of 60 years are said to be particularly affected by it. There is a sense of normalization of visual impairment among the older adults too. It is considered to be a normal process of ageing. A local term ‘*cholisia*’ is used in the study community to refer to the gradual impairment of vision with age. A low vision male study participant in the old-old age group remarked how visual impairment (‘*eye defect*’) is bound to happen with advancing age. This perception is also shared by men and women old-old study participants with high visual impairment.

The symptoms of visual impairment include hazy vision, cataract (‘*chani*’), headache, problems in seeing during the day, problems in seeing during the night, watery eyes, and problems with near and/or far vision. Various words are used by the study participants to denote a symptom. The eyes are ‘*bound to water*’ with advancing age, as was emphatically stated by an old-old woman with high visual impairment. Study participants themselves are likely to have perceived their visual impairment. Gendered idioms of expression have been discerned in the narratives pertaining to symptoms of visual impairment. Study participants often narrate about their symptoms in the context of their gendered activities. For example, difficulties may be reported in the context of sewing for women and outdoor activities for men.

The young-old with low vision do not report much difficulty due to visual impairment. Study participants with high visual impairment, and in the older age groups do. Difficulty in the discharge of regular chores (such as eating, bathing or combing hair) *due* to visual impairment is rarely reported by any of the study participants across the various groups. As a study participant said, ‘*I can do these (regular chores) quite well*’ (Male, Old-Old, Low Vision). Reduced functioning or hardships encountered are not attributed to impaired vision. Rather, they are due to other reasons such as failing health or the external environment. Stumbling and falling on the road, for example, is not perceived to be due to visual impairment. A male study participant explained why tripping while walking was not due to the fault in his eyes. Rather, it was a case of walking on an uneven surface where he accidentally put his foot on a high level (Male, Young-Old, Low Vision).

Participation in economic activities such as sewing, weaving nets or farming may get restricted/ discontinued with impaired vision. But, visual impairment is unlikely to be considered to be debilitating. A woman study participant in the young-old, low vision group recounted how she started using spectacles to continue stitching rugs. Older study participants may have withdrawn from economic activities due to their impaired vision. Women study participants narrated how household members urged them to stop working because of the impairment. One of them said:

*‘They (family members) asked me to stop doing work … weaving nets … because I was not able to see. They told me not to do it (weaving nets) … I am unable to do it because of my eyes … what can I do since I cannot see? … I have stopped doing it'* (Female, Old-Old, Low Vision)

### Eye care seeking behaviour in persons aged 50 years and more

Study participants limitedly access health care facilities for their visual impairment. These range from not having sought any treatment, to a maximum of having visited three health care facilities. Though delayed, outpatient care has been sought by most men and women in every group. The delay may be of months, or even years, after the onset of the symptoms – *‘very long’* as many study participants concede. A male study participant elaborated how he had sought care for blurred vision after a few years. In his words, *‘I used to paint tar … Once tar fell into my eyes and since then I have blurred vision. I visited the doctor three to four years later’* (Male, Young-Old, Low Vision). Visual impairment in older adults is considered to be an inevitable, painless and a non-debilitating condition. As we have seen in the earlier section, it is not considered to be the reason for restriction of the movement or activities of the older adults, especially in cases of low vision. Moreover, seeking medical care is associated with the old and the infirm – a population sub-group that the young-old does not identify with. Distancing herself from such persons, a woman study participant tellingly said that ‘*it is the old who seek care from the doctor*’ (Female, Young-Old, High Visual Impairment). Care seeking for eye health assumes low priority in such circumstances.

Outpatient care was typically sought from nearby health care providers. Such services were usually offered free of charge by the government or NGOs. These may have been offered in the neighbourhood or in a neighbouring locality within the village. Study participants would have walked, cycled or availed of local public transport services to reach the health care facilities offering outpatient care. Services of distant health care providers had been sought by very few study participants for outpatient care. The few who sought such distant care belonged to two groups: young-old with high visual impairment, and middle-old with low vision.

There is a gendered pattern in seeking care for eye health. Mobility and economic independence typically enjoyed by men (especially those in the young-old and the middle-old groups) enabled them to seek care even it meant travelling to distant places. Women study participants visited nearby facilities. The deductive code of ‘Beliefs and Perceptions’ contained narratives explicating prevalent gender norms. Men were more mobile while women were usually dependent on others to take them to (far) places. They were also burdened with household chores. As a woman said, *‘Women are probably afraid, therefore they do not seek services as much (as men). They are afraid because they need to do household work … they do not know what will happen after the treatment (what if they are unable to do household chores? who will do then?) ... Therefore, they are less likely to seek care for their eyes’* (Female, Middle-Old, Low Vision).

The restricted (and often dependent) mobility of women and social norms to be protective towards them are apparent in the narratives of the male study participants. A man narrated how men ‘*can travel (on their own) by bicycle easily. But a woman needs separate transportation to be ferried around … such as a motor cycle, a rickshaw or some other vehicle*’ (Male, Middle-Old, High Visual Impairment). Men are also protective towards the women and tend to accompany them on visits. As a man said, ‘*Perhaps men can tolerate more than women. Or, it may be that we are over protective of them. Therefore, we try to rush to their aid’* (Male, Middle-Old, Low Vision). The combination of women being burdened with household chores, their restricted mobility and the social norm to be accompanied by men results in their limited care seeking for visual impairment.

Inpatient care is limitedly sought, being mostly restricted to men in the young-old and middle-old age groups. Where sought, the study participants are likely to have paid for the expenses themselves. These include hospitalization and travel costs. They would have travelled by bus or other vehicles to the health care facility. Sometimes, they would have availed of free transport services provided by local NGOs to take patients to such destinations. Study participants seeking inpatient care may have been accompanied by others (family members in particular) or would have visited alone (especially in the case of men).

Gender differentials can be discerned in the decision to seek inpatient care. In the case of the ageing women, the decision is taken by other members of the family. Women are also likely to be accompanied by their children or other relatives. Ageing men themselves took decisions on seeking inpatient care and visited health care facilities alone. Access to assets enables the older adults to seek inpatient care. However, it is mediated by gender. Ageing men may take out from their earnings/savings. They may borrow from others to pay for the inpatient services. For the ageing women though, the money for inpatient care is provided by their husbands and/or children. The access to assets and consequently, the seeking of inpatient care for eye health, can be precarious for the ageing study participants of both genders. As a male study participant put it succinctly,

*‘There are families where children own the property forcefully and no longer obey the elderly … then the problem is who will take care of the elderly, who will take him/her, pay the money, this is the problem, there are many such families.* … *if I have property, and wish to visit a certain facility, they are bound to take me there. If you do not have property, even if you beg, they will not take you there’* (Male, Young-Old, Low Vision)

### Decreasing privileges of ageing men

Eye care seeking behaviour changes for men with increase in age and visual impairment. The group of middle-old with high visual impairment and the two old-old groups seek less care. This is true for both outpatient and inpatient care for eye health.

Excepting old-old men with high visual impairment, male study participants are usually engaged in productive activities (such as manual farming). The need for human support has been reported by all male study participants. Such human support may be sought for food, washing of clothes, or purchasing things from the market. Human support is most sought by men in the old-old groups of low vision and high visual impairment. Both groups in that category report needing support for buying medicines or visiting the doctor. Men in all groups report the use of aids in their daily lives. The use of walking sticks has been reported by men in the middle-old and old-old age groups. Taking a lantern or torch while going to the toilet at night has been reported. Ageing men may find it difficult to carry out routine chores such as answering the calls of nature, reading or praying. They may increasingly seek the company of other people for going out. They may restrict outdoor activities in order to be in familiar surroundings. The narratives are telling.

*‘I (no longer) go outside for offering Namaz (Islamic prayer) after dusk. What if I fall down somewhere? I need to bring the book close enough so that I can read’* (Male, Old-Old, High Visual Impairment)

The patriarchal privileges traditionally enjoyed by men decrease with age. Their mobility gets restricted and their economic independence is compromised. It is likely that the old-old may have reduced earnings or would have altogether withdrawn from economic activities. There is an increase in their (physical and economic) dependency on others. Their dominance in the household diminishes. As a male study participant put it, *‘earlier I was the guardian of the household … now my sons have grown up and are earning themselves. I seek money from them’* (Male, Young-Old, Low Vision). The situation is reversed from earlier times when men could support others financially and were often the economic backbone of the household. Seeking care for their eyes is affected in such a situation of reduced privileges with compromised mobility, economic independence and position in the household. One study participant said, ‘*I can easily ask money from my sons, but you know, it is not good to pressurize them for my own benefit. It will look like luxury*’ (Male, Middle-Old, Low Vision). Another man recounted how he did not seek medical care for his eyes because of economic reasons. In his words, ‘*I could not arrange for the money and hence did not go (for getting my eyes treated), my sons will not give the money’* (Male, Old-Old, High Visual Impairment).

### Compounding vulnerability of ageing women

Compared to their male counterparts, female study participants consistently seek less outpatient and inpatient care for eye health. There is no perceptible difference in the seeking of eye care by women across age groups and levels of visual impairment. They are likely to visit the nearby health care facilities for outpatient care for their eyes. Seeking inpatient care for their eyes is limited.

Though sporadically, female study participants of all the three age groups report engagement with household work. Women in all groups (except the old-old with high visual impairment) may be engaged in productive activities such as winnowing grains and cutting fish. Similar to the male study participants, the need for human support has been reported by all women study participants. A woman’s daughter-in-law or grand children may help her in wearing clothes. They may clean her clothes. A person may get water from the tube wells or the ponds so that she may take her bath. Cooking is generally done by the daughter-in-law. A woman said, *‘I no longer cook. I used to do it so well earlier’* (Female, Middle-Old, High Visual Impairment). Another woman recounted how she has stopped cooking for the household and is increasingly dependent on others for routine chores such as bathing and cleaning clothes. She said, *‘It’s been 10-12 years since I stopped cooking (for the household). Who will do my chores for me? Whoever gets some time helps me out’* (Female, Old-Old, Low Vision). With increase in age, things may come to such a pass that food is served to the woman, she being typically dependent on her daughter(s)-in-law for it.

The economic dependence of the female study participants continues into old age. Never self-reliant, they need to take money from their husbands and/or sons for meeting expenses. Two female study participants (both in the young-old age group) narrated how they were economically dependent on their sons. In their words:

*‘Whenever there is a need to buy something or to spend something somewhere, my younger son bears the expenses’* (Female, Young-Old, Low Vision)

*‘My elder son is erratic in matters of money. Sometimes he gives, at other times he does not. He may suddenly give some money after six to nine months’* (Female, Young-Old, High Visual Impairment)

Support needed for buying medicines or visiting the doctor is not as pronounced in the narratives of the female study participants. This may possibly be due to the fact that women typically seek outpatient care from nearby facilities. They are usually accompanied by other (male) members of the family who bear such expenses. Women across the different groups report the use of aids such as walking sticks and torches. As in the case of the male study participants, the use of walking sticks is especially reported from the age group of middle-old onwards. Physical dependency increases with age induced debility. The traditional gendered roles of doing housework (e.g. cooking and cleaning) and child care get reduced with increasing age. Their economic dependence continues into old age. The vulnerability of women gets compounded with age.

## Discussion

The foregoing section presents a granulated picture of health care seeking for visual impairment by ageing men and women in the Indian Sundarbans. Gendered privileges and vulnerabilities play out differently with advancing age and levels of visual impairment. To our knowledge, such an explication of the intersection of gender, age and visual impairment on eye care seeking behaviour is new to scientific literature. This article also presents a methodological innovation in the empirical exploration of intersectionality. The sampling design customizes existing principles of qualitative research sampling (purposive sampling, extreme case sampling) to present a sample that captures the intersecting characteristics of gender, age and visual impairment.

The young-old with low vision have perceived low severity of their visual impairment. They also do not identify themselves with the ageing, infirm population. This may explain why care seeking for eye health is low for the group. For the other groups of study participants, a combination of factors results in the manifest eye care seeking behaviour. Unlike physical infirmity, visual impairment is not considered to be debilitating. There may be gradual withdrawal from economic activities. Carrying out routine activities may get increasingly challenging. However, study participants are likely to attribute their dependency to decreased physical capacity rather than impaired vision. Visual impairment is perceived to be inevitable in old age. It is a painless condition too. With increasingly restricted activities within and outside the house, seeking of care for impaired vision assumes low priority. There is delayed care seeking for visual impairment.

There are distinct gendered patterns in the economic independence and mobility of the study participants. Men are most mobile in the young-old and middle-old age groups. Their mobility decreases as they grow older. With increasing age and visual impairment, their dependency on human and non-human support increases. Their economic independence is often compromised and their position in the household diminishes. This results in lessened care seeking for eye health by men for both outpatient and inpatient care. The patriarchal privileges that they traditionally enjoy are offset by advancing age and visual impairment. Coston and Kimmel observe that male privileges can be ‘compromised’ by disability status, sexuality and class. They term such situations as ‘marginalized masculinities’ [18]. Women, on the other hand, are generally restricted in their care seeking for visual impairment. They typically seek nearby outpatient care. Neither economically self-sufficient nor independently mobile, they gradually withdraw from traditional gender roles such as cooking, cleaning and childcare. Advancing age and visual impairment compound their vulnerability. With increasing dependency on human and non-human support, seeking eye care remains limited for them as they age.

Increasing dependency with the advancement of age in both men and women explains why narratives on eye care seeking are densest for two groups - the young-old with high visual impairment, and the middle-old with low vision. In both genders, these two groups have perceived severity of their visual impairment and are generally able to seek care. The gendered nature of power plays out therein. The differential nature of ‘who has, can acquire, and can expend assets and decisions over one’s body’ [15] shapes the experiences of men and women in seeking care for their visual impairment. This article thus contributes to a layered understanding of access to eye care. Dimensions such as geographic and financial accessibility are known to be gendered, with women being particularly disadvantaged. However, as our results show, access to eye care can be challenging for both men and women with age related infirmity, increasing dependency and change in socio-economic status with advancing age. Our study provides evidence to revisit existing conceptualizations on access to health care [e.g. 6, 19] and add/refine elements that are reflective of the complex contexts of the people in need of care. In a recent conceptualization of patient-centred access to health care, Levesque and colleagues define ‘access as the opportunity to identify healthcare needs, to seek healthcare services, to reach, to obtain or use health care services and to actually have the need for services fulfilled’. They ‘conceptualise five dimensions of accessibility’. These are: approachability; acceptability; availability and accommodation; affordability and appropriateness. The authors say that the five dimensions of accessibility ‘interact’ with five corresponding abilities of populations ‘to generate access’. The five corresponding abilities are: ability to perceive, ability to seek, ability to reach, ability to pay and ability to engage [19]. Our study shows that while differences exist between the groups, the five corresponding abilities change and usually get compromised with advancing age in the context of eye care. The intersectional manner in which study participants experience their lives adds further complexity to the conceptualization formulated by Levesque and colleagues. It calls for greater recognition of the situatedness of the population groups in need of care.

Some findings of the present article corroborate with existing scientific literature. Lack of perceived need/low need has been seen to be a major reason why people with visual impairment do not access eye care services in India [20] and neighbouring Pakistan [21]. Multiple barriers have been cited in accessing eye care services in India [22]. Though women are more likely to be affected by cataract, men tend to receive more services pertaining to it (ibid). Worldwide too, women have restricted access to eye care services. These are due to multiple factors, ranging from ‘sociocultural influences and financial limitations to education level of the patient and awareness of the care provider’. Transportation is a common barrier for women [23].

However, the manifestation of the intersecting characteristics of gender, age and visual impairment in eye care seeking is little understood. The concept of intersectionality receives a synergistic boost as equity in health gains traction in academics, policy making and programme implementation. Larson and colleagues observe that applications of intersectionality in public health has ‘mostly increased’ after 2009 [10]. They identify 86 articles, only 14 of which are on low- and middle- income countries. Thus, this article makes methodological and empirical contributions to intersectionality in public health, in the particular context of a lower middle income country such as India.

The recently released World Report on Vision [24] proposes Integrated People-Centred Eye Care (IPEC) as an approach to strengthen health systems to provide eye care through the life course. The four strategies of IPEC are: (i) engaging and empowering people and communities (ii) reorienting the model of care based on a strong primary care (iii) coordinating services within and across sectors, and (iv) creating an enabling environment, specifically the inclusion of eye care in national health strategic plans, the integration of relevant eye care data within the health information systems, and the planning of the eye care workforce according to population needs. The renewed commitment to primary care in IPEC is welcome as it holds the promise to provide eye care that is available, accessible and sensitive to local needs. It would mean responding to the needs of older adults in their complex contexts.

Intersectional experiences are shaped by numerous characteristics and identities. The present article is restricted to three characteristics (gender, age and visual impairment) in order to exemplify how intersectionality shapes eye care seeking behaviour in a disadvantaged region of India. Though we have studied some more characteristics, data suggested that these three have been crucial in the seeking of eye care. The restriction to three characteristics marks a limitation since, theoretically, more characteristics and identities could have been taken into consideration in the empirical exploration of intersectionality. This limitation can be overcome by subsequent empirical research where a wider range of characteristics and identities is expected to provide an even more granulated picture of intersectional experiences and care seeking behaviour.

There is another limitation of this study. Narratives of the study participants constitute the empirical basis of this article. While they are rich and revealing of the intersectional experiences, we do not know if care seeking for eye health has been appropriate. The study participants have poor literacy status and are based in the Indian Sundarbans. Their perceptions of need and what constitutes care also determine the resultant care seeking behaviour. The narrative accounts are telling but need to be complemented by systems records of the quality of care received and the adequacy thereof. This is beyond the scope of our study, restricted as it is to the understanding of eye care seeking behaviour of older adults in the Indian Sundarbans.

## Conclusions

The health of older adults gains attention as populations age in countries across the world. The socio-demographic diversities of the older adult population necessitate a granulated understanding of their situation. There is a need for consciously veering away from the monolithic construction of such a large population subgroup in any given society. This article has sought to bring forth such a nuanced understanding of the ageing population in India, by highlighting their intersectional existence and its effect on eye care seeking behaviour. Realizing universal access to health care for such a population subgroup is critically dependent on a sound understanding of the variegated contexts of their lives.

## Supplementary information

**Additional file 1.** Annexure 1: COREQ Checklist.

## Data Availability

The dataset is available from the corresponding author on reasonable request.

## Abbreviations

COREQ: Consolidated Criteria for Reporting Qualitative Research
GAF: Gender Analysis Framework
IPEC: Integrated People-Centred Eye Care
NGO: Non-governmental Organization

## Footnotes

1 National Programme for Control of Blindness, Government of India [13] defines the five categories of visual impairment as follows: Normal Vision: > 6/18 in both eyes; Low Vision: < 6/18–6/60 in the better eye; Economic Blindness: < 6/60–3/60 in the better eye; Social Blindness:< 3/60 in the better eye; One Eye Blindness: < 6/60 in one eye and better than 6/18 in the fellow eye; Blind: < 6/60 in the better eye.

2 The criteria of gender and age remained unchanged during the purposive selection of the cases for the qualitative sample. There were very few cases of one eye blindness in the ageing visually impaired population in the study sites. Therefore, cases of social blindness were taken in villages where those of one eye blindness could not be found (within the given gender and age criteria). Similarly, when cases of social blindness could not be found, then persons with economic blindness were included in the sample. In the article, high visual impairment refers to cases with any one of these three eye conditions (economic/social/one eye blindness).
