# Supplementary material for: How does eye care seeking behaviour change with increasing age and visual impairment? Intersectional analysis of older adults in the Indian Sundarbans
Source: BMC Geriatr. 2020 Feb 18;20:71. doi: 10.1186/s12877-020-1438-y (PMC7029516; doi:10.1186/s12877-020-1438-y)
Supplement: Supplementary file 1 — Additional file 1. Annexure 1: COREQ Checklist. [file 12877_2020_1438_MOESM1_ESM.docx]

**Manuscript Title:**

How Does Eye Care Seeking Behaviour Change with Increasing Age and Visual Impairment? Intersectional Analysis of Older Adults in the Indian Sundarbans

**Authors:** Debjani Barman, Manasee Mishra

**Annexure 1: COREQ Checklist**

| **No.** | **Item** | **Guide Question/ Description** | **Response** |
| --- | --- | --- | --- |
| **Domain 1: Research Team and Reflexivity** | | | |
| *Personal Characteristics* | | | |
| 1. | Interviewer/  facilitator | Which author/s conducted the interview or focus group? | Lead author |
| 2. | Credentials | What were the researchers’ credentials? | PhD |
| 3. | Occupation | What was their occupation at the time of the study? | Faculty members in a university |
| 4. | Gender | Was the researcher male or female? | Female |
| 5. | Experience and training | What experience or training did the researcher have? | The researchers have received professional training on qualitative research. They have carried out several qualitative studies. |
| *Relationship with Participants* | | | |
| 6. | Relationship | Was a relationship with the participants established prior to study commencement? | Yes, during the preceding stages of optometric tests and quantitative data collection. |
| 7. | Participants’  knowledge | What did the participants know about the researcher? | Participants knew about our institutional affiliation and contact details. They knew that we were carrying out research and our reasons for doing it. They knew that the lead author was in charge of the study. |
| 8. | Interviewer characteristics | What characteristics were reported about the interviewer/facilitator? | The participants were informed about our institutional affiliation and contact details. They were informed that we were carrying out research and the reasons for undertaking it. They were informed that the lead author was in charge of the study. |
| **Domain 2: Study Design** | | | |
| *Theoretical Framework* | | | |
| 9. | Methodological orientation and theory | What methodological orientation was stated to underpin the study? | Ethnography |
| *Participant Selection* | | | |
| 10. | Sampling | How were participants selected? | Purposive sampling of extreme cases |
| 11. | Method of approach | How were participants approached? | Face-to-face interviews, followed by telephonic interviews |
| 12. | Sample size | How many participants were in the study? | 24 |
| 13. | Non-participation | How many people refused to participate or dropped out? Reasons | None during the face-to-face interview phase. There was one refusal during the telephonic interview phase. The reason is not known. |
| *Setting* | | | |
| 14. | Setting of data collection | Where was the data collected? | Participants’ homes |
| 15. | Presence of non-participants | Was anyone else present besides the participants and the researchers? | Rarely |
| 16. | Sample description | What were the important characteristics of the sample? | Participants were 50 years and more in age, could be male or female, and had varying degrees of visual impairment. They resided in an impoverished region of India. |
| *Data Collection* | | | |
| 17. | Interview guide | Were questions, prompts, guides provided by the authors? Was it pilot tested? | Yes, the study tool was provided by the authors. Pilot testing has been done. |
| 18. | Repeat interviews | Were repeat interviews carried out? If yes, how many? | Yes, twelve |
| 19. | Audio/visual recording | Did the research use audio or visual recording to collect the data? | Yes, audio recording was done. |
| 20. | Field notes | Were field notes made during and/or after the interview or focus group? | Yes |
| 21. | Duration | What was the duration of the interviews or focus group? | Approximately one hour and 20 minutes |
| 22. | Data saturation | Was data saturation discussed? | Yes |
| 23. | Transcripts returned | Were transcripts returned to participants for comment and/or correction? | No |
| **Domain 3: Analysis and Findings** | | | |
| *Data Analysis* | | | |
| 24. | Number of data coders | How many data coders coded the data? | Two |
| 25. | Description of the coding tree | Did authors provide a description of the coding tree? | Yes |
| 26. | Derivation of themes | Were themes identified in advance or derived from the data? | The themes were derived from the data |
| 27. | Software | What software, if applicable, was used to manage the data? | NVivo 10 |
| 28. | Participant checking | Did participants provide feedback on the findings? | No |
| *Reporting* | | | |
| 29. | Quotations presented | Were participant quotations presented to illustrate the themes/ findings? Was each  quotation identified? | Yes |
| 30. | Data and findings consistent | Was there consistency between the data presented and the findings? | Yes |
| 31. | Clarity of major themes | Were major themes clearly presented in the findings? | Yes |
| 32. | Clarity of minor themes | Is there a description of diverse cases or discussion of minor themes? | Yes |
